# Prevention of anxiety and depression in the age group of 75 years and over: a randomised controlled trial testing the feasibility and effectiveness of a generic stepped care programme among elderly community residents at high risk of developing anxiety and depression versus usual care [ISRCTN26474556]

**DOI:** 10.1186/1471-2458-6-186

**Published:** 2006-07-18

**Authors:** Nelleke van 't Veer-Tazelaar, Harm van Marwijk, Patricia van Oppen, Giel Nijpels, Hein van Hout, Pim Cuijpers, Wim Stalman, Aartjan Beekman

**Affiliations:** 1 Department of General Practice, VU University Medical Centre, Amsterdam, The Netherlands; 2 Institute for Research in Extramural Medicine, VU University Medical Centre, Amsterdam, The Netherlands; 3 Department of Psychiatry, VU University Medical Centre, Amsterdam, The Netherlands; 4 Department of Clinical Psychology, VU University Medical Centre, Amsterdam, The Netherlands

## Abstract

**Background:**

In frail elderly, the effects of depression and anxiety are deep encroaching. Indicated prevention studies, aimed at subjects with subthreshold disorder, have shown that well designed interventions are capable of reducing the incidence of depression and anxiety. In this randomised prevention trial for elderly, living in the community and suffering from subthreshold depression and anxiety, a stepped care programme was put together to be tested versus usual (GP) care.

**Methods/design:**

Design: randomised controlled trial.

(*See *figure 1: *organisation chart*) together with two other projects, this project is part of a national consortium that investigates the prevention of anxiety and depressive disorders in later life using a stepped care programme. The three projects have their own particular focus. This project is aimed at elderly living in the community.

Inclusion: subjects with a high risk for depression and anxiety without clinical evidence of these syndromes. The participants are 75 years of age and over and have subthreshold symptoms of depression and or anxiety: they score above the cut-off point on the self-report Centre for Epidemiologic Studies Depression (CES-D) scale, but the criteria for a major depressive disorder or anxiety disorder (panic disorder, agoraphobia, social phobia, generalized anxiety disorder) according to a validated interview, the Mini International Neuropsychiatric Interview (MINI) are not fulfilled.

Outcomes:

primary outcome: incidence of a depressive or anxiety disorder over a period of two years (MINI);

secondary outcome: a positive influence of the intervention, a stepped care programme, on symptoms of depression and anxiety and on quality of life as assessed with the CES D, the HADS A and the SF36 respectively (i.e. stabilisation or improvement of symptoms) [see table 1].

Measurements:

Take place at baseline and at 3, 6, 9, 12, 18 and 24 months. Trained independent evaluators assess depression and anxiety status, the primary end point (6, 12, 18, 24 months) [see table 2].

**Discussion:**

Late-life depression and anxiety are characterised by high prevalence, unfavourable prognosis, reduced quality of life, excess mortality and substantial societal costs. No health service, however well equipped, will be able to effectively treat all elderly with depression and anxiety. Therefore, development of (cost) effective means to prevent these disorders is very important.

## Background

Depression and anxiety lead to a serious impairment of daily functioning and quality of life. In frail elderly, the effects of depression and anxiety are especially deep encroaching. Besides a deleterious effect on daily functioning and quality of life, a large number of studies demonstrate excess mortality, disability, handicap and service utilisation [1-7].

The number of elderly is rapidly growing. Almost a third of elderly subjects in the community with subthreshold depression or anxiety will develop a major depressive or anxiety disorder in three years [8]. Therefore, preventing the onset or development of these disorders has a high priority [9,10]. Three types of prevention can be distinguished; universal prevention (aimed at the general population regardless of risk status), selective prevention (aimed at high-risk groups), and indicated prevention (aimed at subjects who have early symptoms of a disorder but do not meet diagnostic criteria). The aims of these types of preventive intervention are reduction of occurrence of new cases and the delay of onset of illness. Additionally, the aims of indicated preventive interventions might be to shorten the duration of the persistence of the early symptoms and to stop the progression of severity so that the subject does not meet DSM IV diagnostic levels [11]. Indicated prevention studies have shown that well designed interventions are capable of reducing the incidence of depression and anxiety [12]. Moreover, they are likely to be more cost-effective than alternative approaches [13]. So, favourable prevention should be well designed and should be aimed at selected groups of elderly, at high risk of developing anxiety or depression. Selected risk factors for the onset of late life depression are [10]: loss of a partner, chronic illness, neuroticism, family history, lack of social support and subthreshold anxiety or depression disorders.

This project aims at the latter; the design and testing of a stepped care intervention to prevent the onset of full blown anxiety and depressive disorder among elderly people with subthreshold anxiety or depression disorder.

The chosen intervention is a stepped care programme [14-16]. In an environment of limited resources, it makes sense to provide expertise, and all the time and individual attention a patient needs, but not more. Stepped care models represent endeavours to maximize the effectiveness and efficiency of decisions about allocation of resources in therapy. Not all elderly with subthreshold anxiety or depression need the same type and intensity of preventive intervention. Frequently, complaints will disappear without active intervention. Some may be helped by reading a self-help book or watching an instructional video. Others could benefit from a brief psycho-educational group conducted by a paraprofessional or problem solving treatment, and still others may require a form of pharmacotherapy or more intensive individual psychological treatment. Although stepped care seems a logical approach from the clinical perspective, surprisingly few studies have actually examined the effects of stepped care programmes.

The proposed generic stepped care programme is based on the following four assumptions: (a) empowerment; participants with subthreshold anxiety and depression need personalised, repeated education, counselling and confrontation with their symptom levels to be able to acknowledge them, (b) different persons require different levels of preventive activities; (c) determining the right level of preventive intervention is critically dependent on proactively monitoring outcome; and (d) stepping up from lower, less intensive to higher, more intensive levels of preventive activities based on monitored outcomes may increase effectiveness and lower costs overall.

## Methods/design

### Aim of the study

a) To put together an indicated prevention intervention for participants 75 years of age and over who live in the community and suffer from subthreshold anxiety or depression (but have no evidence of the clinical disorders) for use by home care in collaboration with mental health care, and

b) To evaluate the effects of this programme versus usual general practitioner (GP) care in the prevention of depressive or anxiety disorders.

### Study design

**Figure 2:**
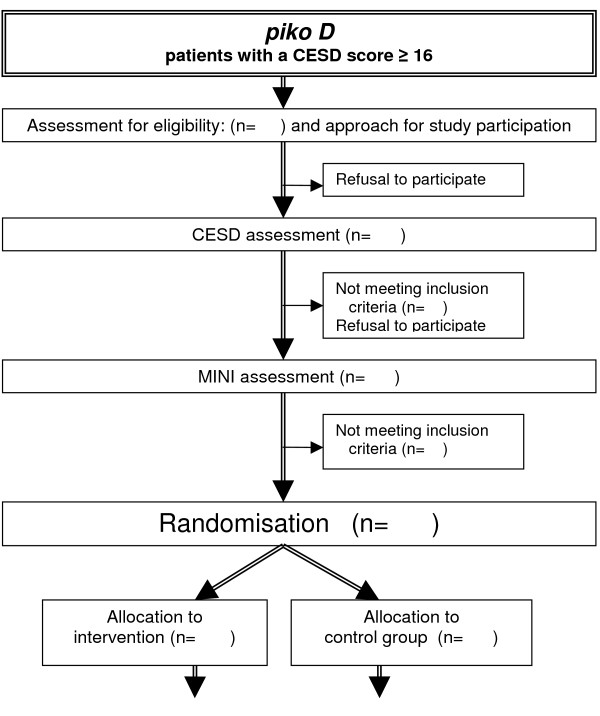
Flowchart

(*See *figure 2: *flow chart*) The study is a prospective randomised two-armed intervention study. Subjects are randomised by an independent statistician and assigned to intervention (stepped care programme) or control group (care as usual).

### Participants

Adult persons, aged 75 years or older, with subthreshold anxiety or depression capable to give informed consent and with sufficient knowledge of the Dutch language are eligible. Subjects meeting criteria for major depression and/or clinical anxiety, subjects with insufficient mastery of the Dutch language and subjects unwilling or unable to give informed consent are excluded. A subject is defined to have subthreshold depression and/or anxiety when he or she scores above the cut-off score of 16 or more on the Centre for Epidemiologic Studies Depression scale (CES-D, a selfrating inventory for depression and/or anxiety), but does not meet criteria for a full-blown depressive or anxiety disorder as assessed with the Mini International Neuropsychiatric Interview (MINI) [17].

### Sample size

Prospective data derived from the Longitudinal Ageing Study Amsterdam (LASA) study show that 27% of elderly subjects in the community with subthreshold depression develop a major depressive disorder within three years [8]. We assume that this percentage is comparable for subthreshold anxiety, and for combined anxiety and depression. The expected incidence rate of DSM-IV depressive or anxiety disorder within two years is estimated conservatively at 35%. Based on previous work in indicated prevention [12,18], we expect the stepped care programme to reduce the incidence rate of major depression and/or anxiety disorder from 35% to 20%. We aim for 110 participants in each arm (α = 0.05 and a power of (1-β) = 0.80). If after final inclusion the total of participants unexpectedly falls short of the required number to reach a power of 0.8 in our calculations, pooling with the two other projects (see organisation chart) will be considered.

### Recruitment

Eligible subjects for the present study are identified among the study population of the larger project for frail elderly -the PIKO project [19] in which this study is embedded. This PIKO project is based on the results of a self-rating health inventory, including the Centre for Epidemiologic Studies Depression scale (CES-D). The inventory has been completed by general practice patients of 75 years or older (see organisation chart). Subjects in this database with a CES-D score above the cut-off point of 16 are approached regarding compliance for the present study.

### Data collection/settings and locations

The data are collected from subjects who live independently on their own in West-Friesland (North-Western part of the Netherlands).

### Outcome measures

The primary outcome is the incidence of major depressive or anxiety (panic disorder, agoraphobia, social phobia and generalised anxiety) disorder (after two years) as measured with the Mini International Neuropsychiatric Interview (MINI)

Secondary outcomes are

-reduction of depressive and/or anxiety symptoms as measured with the CES-D and the HADS-A

-improvement of quality of life (MOS-SF-36)

-mortality

### Instruments

The instruments used in this study are frequently applied in international studies and well validated.

#### MINI diagnostic interview [17,20]

The Mini International Neuropsychiatric Interview (M.I.N.I.) is a short structured diagnostic interview developed in 1990 by psychiatrists and clinicians in the United States and Europe for DSM-IV and ICD-10 psychiatric disorders. With an administration time of approximately 20 minutes, the MINI has become the structured interview of choice for psychiatric evaluation and outcome tracking in clinical psychopharmacology trials and epidemiological studies.

The MINI interviews in this project entail the following modules: depressive disorder, dysthymia, panic disorder, agoraphobia, social phobia and generalized anxiety disorder. For this project we used MiniManager 2.0 (created by E.de Beurs, Leiden University Medical Centre), an electronic version of the Dutch MINI interview (5.0). Both the intervention and control participants will receive blinded independent assessments by telephone of depression and anxiety status at baseline, 6, 12, 18 and 24 months.

#### CES-D [21-24]

The Centre for Epidemiologic Studies Depression scale (CES-D) will be used for the screening of subthreshold depression and anxiety. It consists of 20 items and its total score has a range between 0 and 60. Scores = 16 indicate clinically significant levels of depressive symptoms. At this cutoff the sensitivity is 100% and the specificity is 88% for major depressive disorder in the elderly Dutch population [22]. The CES-D has also been found to be a satisfactory screener for anxiety disorders [23]. However, as the CES-D was designed specifically for the screening of depression and as the criterion validity for depression was considerably better than for anxiety disorders, for follow-up purposes, the CES-D will be combined with additional items from the Hospital Anxiety and Depression scale – anxiety section (HADS-A) [25]. The aim is to optimise the sensitivity and specificity for both subthreshold depression and anxiety.

#### HADS [25]

The Hospital Anxiety and Depression Scale has been found to perform well in assessing the symptom severity and caseness of anxiety disorders and depression in both somatic, psychiatric and primary care patients and in the general population. Of the HADS, in this project only the seven anxiety items will be used to complement the anxiety-screening ability of the CES-D questionnaire.

#### SF (Short Form) 36 – quality of life [26]

The SF36 is the short form questionnaire to measure quality of life. It was used in the original health inventory of the 'embedding' PIKO study.

#### TiC-P health care utilisation [27]

Trimbos/iMTA questionnaire for Costs associated with Psychiatric Illness (TiC-P). This questionnaire will be applied to monitor costs. It is developed by the Trimbos Institute Utrecht in combination with the institute for Medical Technology Assessment Rotterdam

#### Institution of Mental Health – thermometer (Geestelijke GezondheidsZorg thermometer)

An instrument to measure patient treatment satisfaction. It focuses on the appreciation of treatment explanation, the social worker and the result of the coaching.

table 1 displays the outcome parameters, the above mentioned interview and instruments and additionally used questionnaires

### Assessments

Assessments take place at baseline (T0), start of the programme (T1), at three (T2), six (T3), nine (T4) and twelve months (T5) and then at eighteen = follow-up1 (T6) and twenty-four = follow-up2 (T7) months. See table 2

### Intervention/the stepped care approach

The stepped care approach in this project is based on several protocols with known effectiveness [14-16] and discussions with the local homecare agency, regional mental health organisation and other careproviders such as GPs. Two interventions with a strong emphasis on psycho-education and the acquisition of skills with respect to the prevention of depression will be tested. First; the group course "Coping with Depression" (CWD) [28,28-30] which is revised for anxiety and adapted for this specific population (75 years of age and older) and individual use and second; Problem Solving Treatment, which is a brief cognitive behavioral therapy (PST) [31]. Both intervention protocols have come about in collaboration with the Trimbos Institute (the Netherlands) and the Mynors-Wallis (UK) group respectively. A valuable contribution to evidence-based treatments in the elderly might be constituted by the present study in the production of empirically supported treatment protocols.

The stepped care programme as applied in this study entails:

*Step 1: Elderly with subthreshold anxiety or depression are actively followed-up by watchful waiting whether participants recover spontaneously*.

Subjects with a previously high CESD-score are invited to complete a second CESD questionnaire. The interval between the two CESD measurements is at least three months. In case of another score higher than the cut-off score, the MINI diagnostic interview takes place. When the result of the interview is negative (no symptoms of depressive/anxiety disorder during the past year) and the additional inclusion criteria are met, subjects are randomised.

*Step 2, after randomisation. When spontaneous recovery does not occur, participants with subthreshold illness receive minimal interventions (folder, self-help book "Coping with Depression") This step is coached by home care nurses*.

Subjects receive a telephone call with explanation about the intervention. After that, they receive the first questionnaire together with again -but this time; written- information. Shortly afterwards subjects are seen by a specially trained home care nurse who hands over a folder with information about and tips how to deal with subthreshold anxiety and/or depression. The aims of this first visit are: a) to assess type, the level of severity and causes of symptoms, b) to reflect on emotional symptoms through an initial brief nondirectional exploration and discussion of everyday problems, which also helps to establish a working relationship. c) to educate participants about their symptoms. The nurse makes an appointment for the next visit.

During the second visit the folder will be briefly discussed and a reader, the self-help course "coping with depression" will be supplied to the client. Subjects are stimulated to do the course in their own tempo. The nurse will regularly ring or visit the client to discuss progress and questions. Obviously to read both folder and reader and make the exercises offered in the latter, is not obligatory. Subjects are free to read the information and make the exercises. An important aspect of the role of the nurse is advice and encouragement. After three months, at the end of the first phase an evaluation form is completed by the nurse.

Step 3. When symptoms persist, participants are introduced to a brief cognitive behavioural therapy intervention (Problem Solving Treatment)

If -after three months- there is still a CES-D score of 16 or higher, subjects will receive a telehone call in which is explained to them that they now qualify for Problem Solving Treatment. The method and approach are explained to them. The short-term Problem Solving Treatment is given by specially trained Community Psychiatric Nurses, who will also complete an evaluation form at the end of the maximum 7 sessions.

*Step 4. At this final step, participants who still suffer from subthreshold depression or anxiety, are referred to their GP to discuss the appropriateness of specific medication*.

In case of a continuously elevated CES-D score, subjects will receive written advice to discuss suitable medication with their GP (i.e., antidepressants).

Summarising, the CES-D score is checked every three months during a year. Subjects with a result at or above the cut-off score of 16 are offered participation in the next step. A result below the cut-off score means; 'wait and see' for three months, after which a new evaluation takes place. At that point, again, a CES-D score of 16 or higher means that participation in the next step is offered after all. So, in the programme, postponement or cancellation of steps is possible. When a major depression or an anxiety disorder develops, subjects are referred back to their GP.

## Discussion

Late-life depression and anxiety are characterised by high prevalence, unfavourable prognosis, reduced quality of life, excess mortality and substantial societal costs [13]. Fortunately, there are hopeful indications that the prevention of new cases of mental disorders seems to be possible [12]. Subthreshold anxiety and depression (i.e., the presence of symptoms of anxiety or depression without evidence of the actual psychiatric disorder) are prognostic variables for major anxiety disorder and depression [13]. Interventions in the subthreshold disorders may prevent the onset of new cases of major depression and anxiety disorder [18]. Demonstration projects of shared care models for indicated prevention of new cases of mental disorders may be an important step forward to reduce the enormous burden of these disorders as was shown by a recent meta-analysis of randomised trials of preventive interventions [12] In this study the indicated prevention of depression and anxiety is attempted by way of a stepped-care programme offering the aged subthreshold depressed or anxious participant, several interventions ranging from noncommittal to the requirement of some engagement.

A preliminary reflection on the limitations and strengths of our design:

A limitation of this pragmatic design is that it will be difficult to weigh the specific contributions of the various elements of this project. Another limitation may be that the quantity and type of questions (questions about mood may be confronting and depressing on their own) can lead to drop-out. The stimulating ideas and activities in the self-help course trying to achieve a positive and active attitude may have an opposite effect since the reader might instead be confronted with his/her limitations.

Strong aspects of this design:

- It is a unique practice-based project in which several evidence-based aspects of for instance the highly successful IMPACT treatment study (Improving Mood: Promoting Access to Collaborative Care Treatment) [16] are incorporated, now for indicated prevention purposes and among a very old population. Where IMPACT provides its participants of 60 years of age or older with education, brief psychotherapy, medication and stepped care while outcomes and progress are regularly and closely monitored by a care manager, in this Dutch project the participants are older but very similar items compared to the IMPACT model are employed. However, the focus now is on symptoms and on indicated prevention, not on treatment of persons who drew up a specific treatment contract with their physician.

- A further strength of our study is that it encompasses anxiety: that even less studies among the elderly have looked at indicated prevention of anxiety [12,32], and at co-morbidity of anxiety and depression [12]. There are several community studies which show that anxiety is as important a problem for elderly as depression [2,3].

The third strength is that we will also be able to reflect on the ethical dilemma of whether or not to include persons in programmes such as these who are at risk (i.e., not participants) but have no explicit request for care. The percentage uptake of the intervention in the various stepped care stages and the participants' preferences will show us whether an intervention aimed at symptoms and not at syndromes connects to the reality of distressed old people in the community. The questions would be whether these elderly are willing to acknowledge distress and to take active steps to feel better.

Strengths and limitations taken into account, the development and research of (cost) effective means to prevent depressive and anxiety disorder in the rapidly growing group of elderly is a matter of the greatest importance.

### Timeframe of the study

See table 2

### Description of risks

To our knowledge, serious risks or undesired effects of completing questionnaires are not reported in the literature. There are no specific risks related to this study.

### Ethical principles

The participation in the study is voluntarily. Participants are informed that they can cancel their participation at any time without disclosing reasons for their cancellation and without negative consequences for their future medical care.

### Vote of the ethics committee

The design and conduct of the study was approved by the ethics committee of VU University Medical Centre, Amsterdam.

### Data security

Confidential information and participant names are secured by the medical confidentiality rules and are treated according to the code of conduct for medical research, developed by the FMWV (the Federation of Biomedical Scientific Societies).

The results of the participant questionnaires are not accessible to the General Practitioners. All study related documents and data are stored on a protected central server of the Institute for Research in Extramural Medicine, VU University Medical Centre, Amsterdam, the Netherlands. Only members of the study have access to the respective files.

## Competing interests

The author(s) declare that they have no competing interests.

## Authors' contributions

NvtV, HvM, PvO perform the study

HvM participated in the draft manuscript of the proposal, the study design

AB and PC participated in the draft manuscript and the conceiving of this study

GN and HvH provided the setting and the coordination of the 'embedding' project PIKO

AB and WS are responsible for the overall supervision

All authors read and approved the final manuscript

## Pre-publication history

The pre-publication history for this paper can be accessed here:



## Figures and Tables

**Figure 1:**
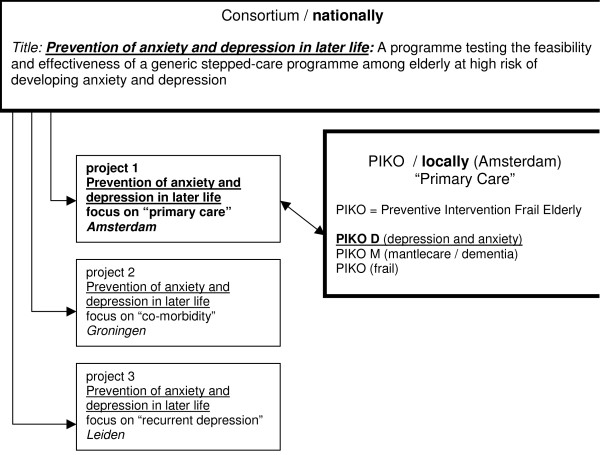
Organisation chart

**Table 1:** Instruments used to establish primary and secondary outcomes

**Outcome measures**	**Instrument**
**Primary outcome**	
depressive/anxiety disorder	MINI diagnostic interview
**Secondary outcome**	
reduction of depressive symptoms	CES-D
reduction of anxiety symptoms	HADS-A
quality of life	MOS-SF-36
health care utilisation	TIC-P
patient satisfaction	GGZ thermometer

**Table 2:** Assessments

	**health inventory T0**	**T1-a**	**T1-b**	**T1-c**	**T2 **3 mnths	**T3 **6 mnths	**T4 **9 mnths	**T5 **12 mnths	**T6 **18 mnths	**T7 **24 mnths
**CES-D**	x	x	-	-	x	x	x	x	x	x
**MINI**		-	x	-	-	x	-	x	x	x
**extra identification information**		-	-	x	-	-	-	-	-	-
**HADS-A**		-	-	x	-	x	-	x	x	x
**SF36**		-	-	x	-	-	-	x	-	x
**TIC-P health care utilisation**		-	-	x	-	x	-	x	x	x
**pt.satisfaction**		-	-	x	x	x	x	x	x	x
